# The jailed balloon technique for preventing coronary obstruction in the high-risk transcatheter aortic valve-in-transcatheter aortic valve

**DOI:** 10.1093/ehjcr/ytag631

**Published:** 2026-08-27

**Authors:** Shuro Narui, Ryosuke Higuchi, Itaru Takamisawa, Mamoru Nanasato

**Affiliations:** Department of Cardiology, Sakakibara Heart Institute, 3-16-1 Asahi-cho, Fuchu, Tokyo 183-0003, Japan; Department of Cardiology, Sakakibara Heart Institute, 3-16-1 Asahi-cho, Fuchu, Tokyo 183-0003, Japan; Department of Cardiology, Sakakibara Heart Institute, 3-16-1 Asahi-cho, Fuchu, Tokyo 183-0003, Japan; Department of Cardiology, Sakakibara Heart Institute, 3-16-1 Asahi-cho, Fuchu, Tokyo 183-0003, Japan

**Keywords:** VIV, Sinus sequestration, Coronary obstruction, Coronary protection

## Case description

Transcatheter aortic valve (TAV)-in-TAV procedures are increasingly common but remain challenging in patients with narrow sinotubular junction (STJ) and/or sinus of Valsalva.^1^

**Figure 1:**
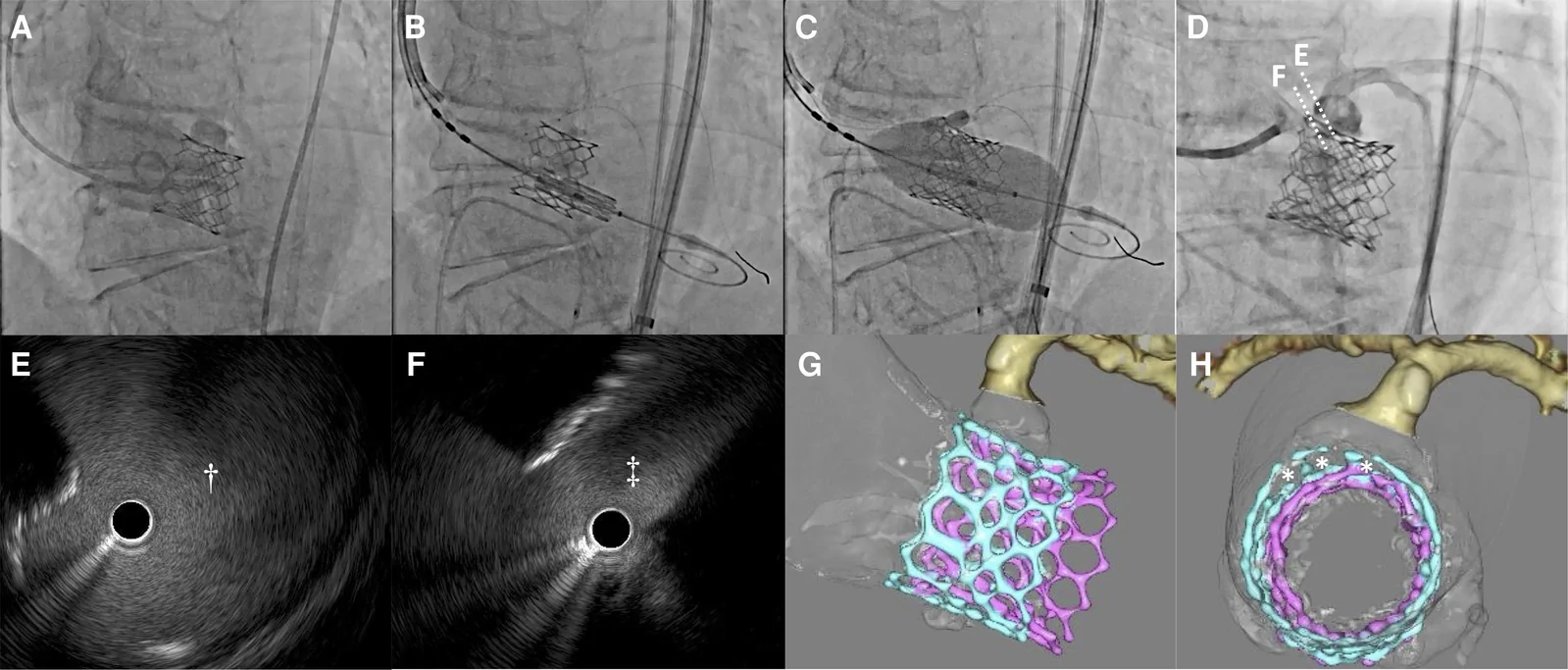
The jailed balloon technique to prevent sinus sequestration. (*A*) Pre-procedural aortography depicting limited sinotubular junction space, (*B*) orthotopic coronary protection, (*C*) valve deployment with simultaneous inflation of a jailed coronary balloon through the initial valve frame into the left sinus, (*D*) preserved sinus flow, (*E*, *F*) Intravascular ultrasound imaging of left sinus (*E*: †), and preserved intervalvular space (*F*: ‡), (*G*, *H*) The second valve implanted at a lower position than the initial valve, with a significant interframe gap(*).

An 87-year-old woman presented with dyspnoea 6 years after TAV implantation with a 23-mm SAPIEN-3 valve (Edwards Lifesciences). Echocardiography revealed severe structural valve deterioration, with an elevated mean gradient of 41 mmHg. Pre-procedural computed tomography (CT) demonstrated that the neoskirt extended above the STJ, with only 0.7 mm valve-to-STJ distance (*Figure 1A*). Considering the limited STJ anatomy, chimney stenting was considered unfeasible, and leaflet laceration was suboptimal because of commissural misalignment. Coronary protection was established orthotopically using a 6Fr Judkins Left 3.5 guiding catheter via the right femoral artery, with guidewires in the left anterior descending and circumflex arteries (*Figure 1B*). A 23-mm SAPIEN3 Ultra RESILIA valve was implanted through the left femoral artery with simultaneous inflation of a 5.0 × 12-mm non-compliant balloon (*Figure 1C*, see Supplementary material online, *Movie*). The jailed balloon restrained the upward displacement of the second valve, creating adequate space between the valve frames (*Figure 1D*). Intravascular ultrasound confirmed sufficient space between the frames, and the final angiography demonstrated preserved sinus flow without the need for bailout coronary stenting (*Figure 1D–F*). Post-procedural CT demonstrated a 3-mm interframe gap, maintaining coronary perfusion (*Figure 1G* and *H*) without evidence of leaflet thrombus. The effective orifice area was 1.9 cm^2^ with no aortic regurgitation detected on transthoracic echocardiography.

In high-risk TAV-in-TAV cases in which conventional coronary protection is not feasible, the jailed balloon technique may be an alternative preventive option against sinus sequestration.

## Supplementary Material

ytag631_Supplementary_Data

## Data Availability

The data underlying this article are available in the article and its online Supplementary material.

## References

[1] Higuchi R, Otaki Y, Kanisawa M, Takamisawa I, Nanasato M, Iguchi N, et al Risk of Sinus sequestration during redo transcatheter aortic valve implantation: the prevalence, predictors, and risk stratification. Am J Cardiol 2024;211:1–8. 37884114 10.1016/j.amjcard.2023.10.058

